# IgM antibodies to oxidized phosphatidylserine as protection markers in cardiovascular disease among 60-year olds

**DOI:** 10.1371/journal.pone.0171195

**Published:** 2017-04-21

**Authors:** Johan Frostegård, Jun Su, Sudhir Sing, Xiang Hua, Max Vikström, Karin Leander, Bruna Gigante, Ulf de Faire, Anna G. Frostegård

**Affiliations:** 1 Unit of Immunology and Chronic Disease, Institute of Environmental Medicine, Karolinska Institutet, Stockholm, Sweden; 2 Department of Emergency Medicine, Karolinska University Hospital, Stockholm, Sweden; 3 Unit of Cardiovascular Epidemiology, Institute of Environmental Medicine, Karolinska Institutet, Stockholm, Sweden; 4 Department of Cardiovascular Clinical Science, Danderyds Hospital, Stockholm Sweden; 5 Department of Cardiology, Karolinska University Hospital, Solna, Sweden; Leibniz-Institut fur Pflanzengenetik und Kulturpflanzenforschung Gatersleben, GERMANY

## Abstract

**Objective:**

Phosphatidylserine is exposed on apoptotic cells and is prone to oxidation (OxPS). Here we analyze the association of IgM antibodies against OxPS (anti-OxPS) with the risk of cardiovascular disease (CVD).

**Methods:**

Among sixty-year olds from Stockholm County in Sweden, previously screened for cardiovascular risk factors (2039 men, 2193 women), there were 210 incident CVD-cases identified during a 5-year follow-up. Using a nested case-control design, 622 age- and sex-matched controls were selected. Odds ratios (OR) with 95% intervals (CI) were calculated by conditional logistic regression. IgM anti-OxPS was measured by ELISA. Phagocytosis of apoptotic Jurkat-cells by macrophages was studied by flow cytometry.

**Results:**

Anti-OxPS levels were lower among cases (median (interquartile range): 80.7 (60.9–101.0 vs. 84.6 (65.8–109.6); p = 0.047); among men (76.6 (55.8–99.2) vs. 82.0 (63.1–105.1); p = 0.022) and among women 89.6 (72.3–110.1) vs. 89.8 (69.9–114.4); p = 0.79).

After adjustment for smoking, BMI, diabetes mellitus type II, hypercholesterolaemia and hypertension, and dividing into quartiles, using the highest quartile (quartile 4) as reference, quartile 3 was associated with a OR of 1.74 (CI 1.08–2.81). Quartiles 2 and 1 had similar associations, the later reaching statistical significance. Among men associations were stronger whereas no significant associations were observed in women. The OR of MI/angina comparing quartile 3 with quartile 4 was 2.31 (CI 1.30–4.11). The OR for quartile 2 and 1, respectively, were similar as for quartile 3.

Total IgM increased uptake of apoptotic cells, which was reversed if incubated with OxPS.

**Conclusions:**

IgM anti-OxPS is a novel potential protection marker for CVD, in particular in men. Increased phagocytosis of dying/dead cells could be one potential underlying mechanism.

## Introduction

Atherosclerosis is the major underlying cause of cardiovascular disease (CVD) such as stroke and myocardial infarction (MI). Atherosclerotic lesions typically contain activated immunocompetent cytokine-producing cells, macrophage-derived foam cells, oxidized low density lipoproteins (OxLDL) and debris/dead cells[1, 2], all of which contribute to inflammation within and destabilization of plaques.

Traditional risk factors for CVD (and atherosclerosis) including age, male sex, hypertension, hyperlipidemia, smoking and diabetes do not account for inflammation and immunity involved in development and progression disease. Several promising inflammatory risk factors are under investigation. High sensitivity C-reactive protein (hsCRP) and IL-6 have been discussed intensively [3], however an inherent volatility associated with these factors might be a limitation for their clinical usefulness at the individual level[2, 4, 5]. Other examples include LDL-PLA2 [6], but like for hCRP, it is not clear whether it inhibits or promotes the underlying atherosclerosis and contributes to clinical events[7]. Further, the independency of these in relation to traditional risk markers is not clear[7].

Among emerging immune factors, our group described earlier IgM antibodies against phosphorylcholine (anti-PC) as independent risk markers of atherosclerosis progression and CVD in the general population and also in patients with systemic lupus erythematosus (SLE)[8–10]. Also IgM antibodies against oxidized cardiolipin were described as independent protection markers in these conditions[2, 11].

Although oxidized LDL cholesterol and inflammatory cytokines and chemokines have been much in focus in atherosclerosis research[2], less attention is given to mechanisms involved in removal of dead/dying cells and cell debris from plaques. Phosphatidylserine (PS) is a phospholipid component of cell membranes and is exclusively present on the inner membrane layer. In events of cellular stress and death, PS is universally relocated to the outer layer, where it plays a key role as a danger associated molecular pattern (DAMP) for recognition by phagocytes. Modification of PS by oxidation appears to be of major importance for phagocytosis of apoptotic cells[12, 13].

To the best of our knowledge, a potential role, including mechanisms involved, of antibodies against OxPS (anti-OxPS) has not been described previously in relation to the risk of CVD events. We here report that IgM anti-OxPS is negatively associated with CVD, especially with stroke in men. One of the proposed mechanisms behind such association is involvement of these antibodies in facilitating uptake of debris/apoptotic cells within the atherosclerotic lesions. The implications of these findings are discussed.

## Methods

### Subjects

From July 1^st^ 1997 to June 30^th^ 1998, every third man and woman in the County of Stockholm, Sweden reaching the age of 60 years, were invited to participate in a health screening for cardiovascular diseases. By this selection of individuals, age bias was thus avoided. A total number of 4232 subjects (2039 men and 2193 women; response rate 78%) participated in the investigation. Information on sociodemography, lifestyle habits, medication and previous diseases and hospitalizations was obtained by a self-administered questionnaire. Physical examination with blood pressure measurements, anthropometry and ECG was performed and serum, plasma and whole blood were collected for storage in a biological bank (-80°C). Details of the screening procedure have been given elsewhere[14, 15]. All subjects gave written informed consent before entering the study. The study including the procedure with written consent was approved by the Karolinska Institutet research ethics committee and is in accordance with the Helsinki Declaration.

### A nested case-control design

To record incident cases of first CVD, new events of coronary heart disease (CHD), defined as fatal and non-fatal myocardial infarction (MI) and ischemic stroke, hospitalization for angina pectoris, were registered. The study base of 4232 subjects was matched with the national cause of death registry (fatal events until December 31, 2003) and the national patient registry (non-fatal events until December 31, 2005). Through these matching procedures 211 incident cases of CVD were recorded. To guarantee that first CVD events were registered, only subjects without a history of CVD prior to recruitment were utilized for the matching procedures. The International Classification of Diseases (ICD-10) was used to register CHD-deaths (I 20, I 21, I 46), MI (I 21), angina pectoris including PCIs and CABGs (I 20, Z 95.5 and Z 95.1) and ischemic stroke (I 63-I 66). The criteria used were thus uniform and consistent. For each case three controls were randomly selected, matched for gender and age (+/- 60 days) at the date of CVD diagnosis. Among these, IgM anti-OxPS-measures were available in 622 controls and 210 cases. Thus, a nested case-control design (210 cases and 622 controls) was applied for the epidemiological and statistical analyses.

### Oxidation of PS

PS was purchased as ethanol solution (Sigma, GmbH, Steinheim, Germany) and was stored at –20°C. PS was oxidized in aqueous solutions containing 1.5 mmol/L tert-butylhydroperoxide and CuSO_4_ in 20 μmol/L essentially as described previously[16, 17].

### Determination of antibodies with ELISA

IgM antibodies to OxPS were determined by enzyme-linked immunosorbent assay (ELISA) essentially as described[16, 17] and in a similar way as IgM antibodies against oxidized cardiolipin.[11] Briefly, serum from a donor with anti-OxPS levels above median levels was used as internal standard and tested on every plate. The plateau of antibody binding was reached with the antigen concentration of 10 μg/mL. Immulon 1B plates (Thermo Labsystems, Franklin, MA, USA) were coated with OxPS (10μg/mL) at 50 μL/well in ethanol and left overnight at 4°C. After five washings with PBS, plates were blocked with 2% BSA-PBS for 2h at room temperature and washed as indicated above. Serum samples were diluted (1:50) in 0.2% BSA-PBS and added at 50 μL/well.

Plates were incubated overnight at 4°C and washed as described above. Alkaline phosphatase conjugated goat anti-human IgM (diluted 1:7000 in the sample buffer) was added at 100 μL/well and incubated at 4°C overnight. After five washings, color was developed by adding the alkaline phosphatase substrate (PNPP) at 100 μL/well and incubating the plates for 60 min at room temperature in the dark. The plates were read in an ELISA Multiscan Plus spectrophotometer at 405 nm. All samples were measured in duplicates and the coefficient of variation was below 15%.

### Phagocytosis of apoptotic cells

CD14^+^ monocytes were cultured in 75mm^2^ flask (Corning Inc, USA) with 50 ng/mL of GM-CSF (Immunotools, Germany). After 3 days of incubation, half of the culture media was removed and same amount of new media was added with GM-CSF. After 6 days CD11b expression was determined by flow cytometry.

Jurkat cells (ATCC^®^ TIB-152, Clone E61) were labeled with tetramethylrhodamine (TAMRA; Sigma-Aldrich, MO, USA) and treated with 0.5 μM staurosporine (Sigma Aldrich, MO, USA) in complete RPMI media for 17 hours. Induction of apoptosis was confirmed by BD Pharmagen kit (BD Bioscience, USA) as per manufacture’s instruction. Finally, apoptotic Jurkat cells were co-cultured with macrophages 5:1 with or without 10 μg/mL IgM (Sigma-Aldrich, Israel), and/or 10 μg/mL OxPS which was either pre-incubated for 90 minutes or simultaneously added in the cell culture system as indicated. Phagocytosis assay was analyzed by flow cytometry.

### Statistics

Data analyses including demographic biochemistry- and anthropometry-related were performed for cases and controls respectively with values expressed as mean ± standard deviations (SD) for normally distributed parameters and medians (ranges) or proportions for parameters which were not normally distributed after logarithmic transformation. Statistical differences between cases and controls were evaluated through non-parametric tests. Odds ratios (OR) with 95% confidence intervals (CI) were calculated applying conditional logistic regression to estimate CVD risk. Analyses were run crude or adjusted for traditional risk factors as indicated. We compared the highest quartile of IgM anti-OxPS values with those below. We also studied how values above or below different cut-off values as described influenced risk.

These analyses were performed using SAS 9.4 release (SAS institute, Cary NC, USA). For all statistical analyses, a two-tailed p-value < 0.05 was considered significant.

## Results

We identified 211 incident cases of first CVD events throughout the follow-up period, 5–7 years, (77 with MI, 85 with angina pectoris and 49 with ischemic stroke). For each incident case three age and sex matched controls were selected, and thus 633 controls. Serum samples were missing for 1 case and 11 controls leaving 210 cases and 622 controls for analyses.

As previously reported in a similar (but not identical) dataset[18], there were more hypertensives and smokers among cases than controls and trendwise higher BMI. Further, blood pressure levels, HDL, hCRP and apolipoproteins were less favourable among cases as compared to controls (Table 1).

**Table 1:** Baseline characteristics among incident CVD cases and matched controls.

	Incident cases	Controls	P value
Number	210	622	NA
Age, years	60	60	NA
Male gender, %	66.2	66.7	NA
Smokers, %	31.9	19.7	0.0007
Diabetes %	24.3	15.8	0.0103
BMI kg/m2	27.7 ±4.7	26.6 ±3.8	0.0022
Hypertension (>140/90 mm Hg), %	42.4	25.6	<0.0001
Glucose mmol/L	6.1 ±2.5	5.6 ±1.5	0.0004
Insulin μmol/L	11.4 ±7.1	10.1±59	0.0176
Systolic blood pressure, mm Hg	148 ±21.8	139 ±21.2	<0.0001
Diastolic blood pressure, mm Hg	89 ±10.6	85 ± 10.4	<0.0001
Cholesterol, mMol/l	6.1 ±1.0	6.0 ±1.2	0.1234
HDL, mMol/l	1.3 ±0.4	1.4 ±0.4	0.0006
LDL, mMol/l	3.9 ±1.2	3.8 ±1.1	0.5304
Triglycerides, mMol/l	1.6 ±1.0	1.4 ±0.8	0.0004
hsCRP, mg/l	2.4 (1.3–4.6)	1.7(0.9–3.2)	<.0001
IgM Anti-OxPS	80.7(60.9–101.0)	84.6(65.8–109.6)	0.047
IgM Anti-OxPS, Men	76.6(55.8–99.2)	82.0(63.1–105.1)	0.0221
IgM Anti-OxPS, Women	89.6(72.3–110.1)	89.8(69.9–114.4)	0.7915

**Fig 1:**
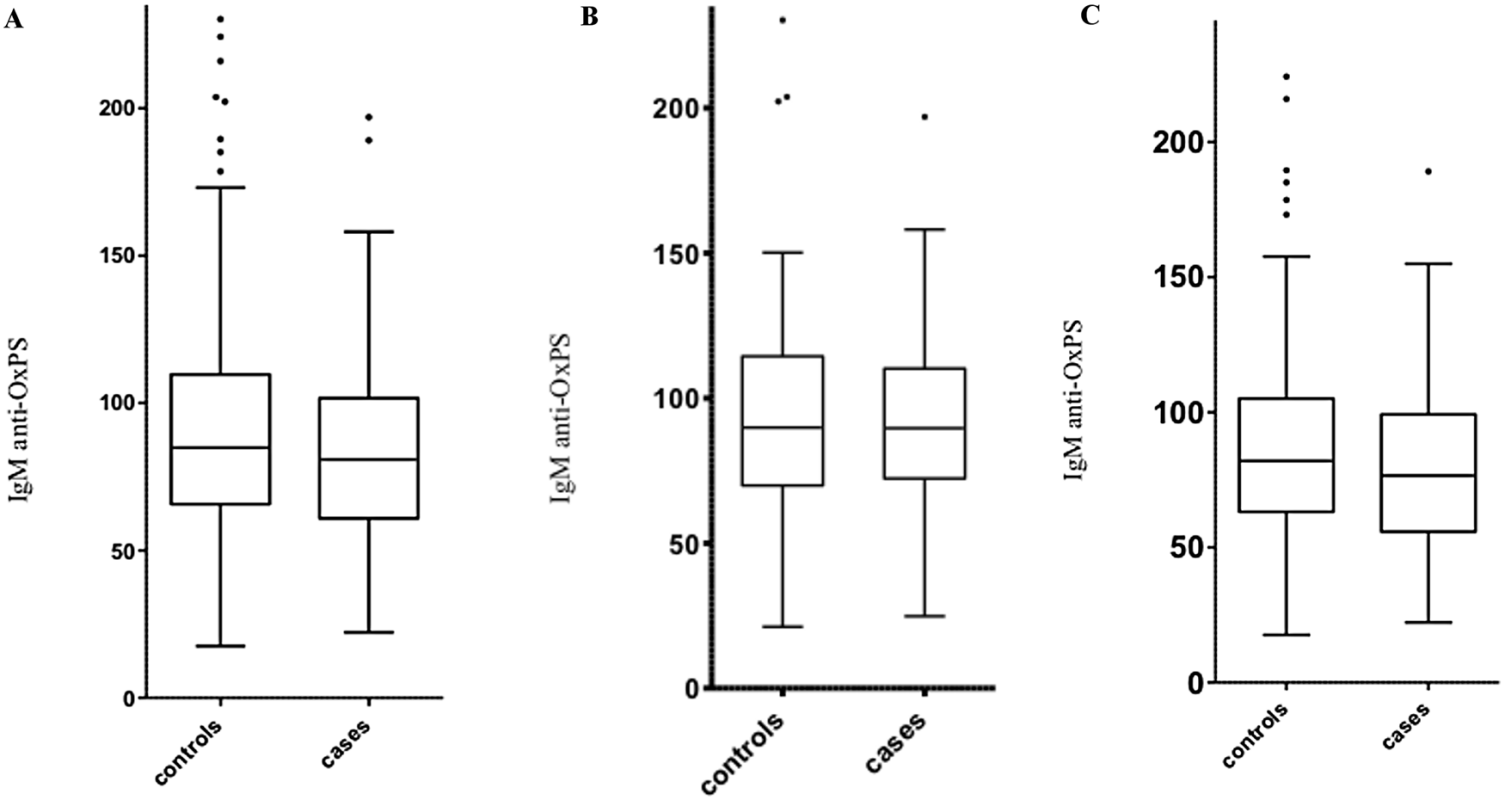
**a. Distribution of IgM anti-OxPS among cases and controls**. 0 = controls who did not develop CVD during the follow up time, 1 = controls who did develop CVD. Cases hade significantly lower levels than controls (p = 0.047). Differences were more pronounced when highest quartile was compared with the other quartiles. **b. Distribution of IgM anti-OxPS among women, cases and controls**. 0 = controls who did not develop CVD during the follow up time, 1 = controls who did develop CVD. Cases hade significantly lower levels than controls (p = 0.047). Differences were more pronounced when highest quartile was compared with the other quartiles. **c. Distribution of IgM anti-OxPS among men, cases and controls**. 0 = controls who did not develop CVD during the follow up time, 1 = controls who did develop CVD. Cases hade significantly lower levels than controls (p = 0.047). Differences were more pronounced when highest quartile was compared with the other quartiles.

Mean values of IgM anti-OxPS were lower among cases than controls (p = 0.047), a difference also present among men (p = 0.022) but not women (p = 0.792; Table 1; Fig 1). IgM anti-OxPS was normally distributed.

After adjustment for smoking, BMI, diabetes mellitus type II, hypercholesterolaemia and hypertension, there was a significant inverse association with risk CVD, as presented in Tables 2 to 6. If IgM anti-OxPS values were divided into quartiles (based on the whole study group), with the lowest set as 1^st^, the 3rd quartile compared to the highest, the 4^th^ had OR 1.74 (CI 1.08–2.81). 2^nd^ and 1^st^ had similar associations, with the lowest 1^st^ reaching significance as compared to the highest.

**Table 2:** Risk of CVD in relation to the highest quartile of IgM anti-OxPS. Association between levels of IgM anti-OxPS and risk for CVD, (as compared to 622 controls) among all participants and men and women separately. In tables, the highest quartile, Q4, is set as 1. Both cases and controls were included as the basis for division into quartiles.

	ALL(210/622)		MALES(139/415)		FEMALES (71/207)	
	Crude OR (95%); P	Adjusted* OR(95%);P	Crude OR (95%);P	Adjusted* OR (95%); P	Crude OR (95%); P	Adjusted* OR (95%); P
Q1	1.73 (1.07–2.18); 0.021	1.77 (1.07–2.91); 0.19	2.25 (1.22–4.15); 0.074	2.47 (1.30–4.72); 0,0043	0.94 (0.39–2.27); 0.89	0.95 (0.39–2.34); 0.913
Q2	1.42 (0.89–2.28); 0.17	1.52 (0.93–2.47); 0.12	1.38 (0.75–2.55); 0.36	1.62 (0.85–3.06); 0.17	1.61 (0.75–3.45); 0.23	1.56 (0.70–3.47); 0.275
Q3	1.63 (1.03–2.59); 0.40	1.74 (1.08–2.81); 0.023	1.83 (0.99–3.37); 0.056	2,04 (1.08–2.86); 0.029	1.42 (0.70–2.92); 0.34	1.47 (0.70–3.10); 0.310
Q4	1	1	1	1	1	1

*adjustment for smoking, BMI, type II diabetes, hypercholesterolaemia and hypertension

(n/n) refers to cases and controls

**Table 3:** Risk of stroke in relation to the highest quartile of IgM anti-OxPS. Association between levels of IgM anti-OxPS and risk for stroke, (as compared to 622 controls) among all participants and men and women separately. In tables, the highest quartile, Q4, is set as 1. Both cases and controls were included as the basis for division into quartiles.

	ALL (49/144)		MALES (25/74)		FEMALES (24/70)	
	Crude OR(95%);P	Adjusted* OR(95%);P	Crude OR(95%);P	Adjusted* OR(95%);P	Crude OR (95%);P	Adjusted* OR(95%);P
Q1	1.90 (0.62–5.84); 0.22	2.78 (0.83–0.34); 0.09	3.29 (0.57–18.93);0.22	8.85 (0.82–95.52); 0.12	1.07 (0.20–5.58); 0.79	1.07 (0.19–6.01); 0.82
Q2	0.78 (0.28–2.17); 0.55	1.02 (0.34–3.05); 0.82	0.85 (0.16–4.56); 0.75	1.79 (0.22–14.89); 0.90	0.85 (0.23–3.15); 0.65	0.88 (0.22–3.54); 0.66
Q3	0.83 (0.32–2.17); 0.67	0.93 (0.32–2.70); 0.81	1.32 (0.27–6.61); 0.87	1.54 (0.21–11.04); 0.74	0.63 (0.18–2.16); 0.71	0.69 (0.18–2.61); 0.85
Q4	1	1	1	1	1	1

*adjustment for smoking, BMI, type II diabetes, hypercholesterolaemia and hypertension

(n/n) refers to cases and controls

**Table 4:** Risk of angina/MI in relation to the highest quartile of IgM anti-OxPS. Association between levels of IgM anti-OxPS and risk for CVD, (as compared to 622 controls) among all participants and men and women separately. In tables, the highest quartile, Q4, is set as 1. Both cases and controls were included angina/MI as the basis for division into quartiles.

	ALL (161/478)		MALES (114/341)		FEMALES (47/137)	
	Crude OR(95%);P	Adjusted* OR(95%);P	Crude OR(95%);P	Adjusted* OR(95%);P	Crude OR(95%);P	Adjusted* OR(95%);P
Q1	1.80 (1.03–3.13); 0.038	1.85 (1.03–3.31); 0.043	2.19 (1.11–4.34); 0.018	2.42(1.17–5.02); 0.043	1.13(0.41–3.11); 0.91	1.24 (0.41–3.80); 0.90
Q2	1.69 (0.96–2.95); 0.056	2.02 (1.12–3.68); 0.018	1.55 (0.77–3.09); 0.22	2.10(1.00–4.41); 0.05	2.25 (0.85–6.01); 0.063	2.55 (0.85–7.74);0.086
Q3	1.94 (1.13–3.35): 0.0099	2.31 (1.30–4.11); 0.0023	2.17 (1.09–4.32); 0.027	2.62(1.26–5.45); 0.011	1.63 (0.66–4.01); 0.16	2.31 (0.79–6.73); 0.068
Q4	1	1	1	1	1	1

*adjustment for smoking, BMI, type II diabetes, hypercholesterolaemia and hypertension

(n/n) refers to cases and controls

**Table 5:** Association between levels of IgM anti-OxPS and risk for MI and/or + stroke (CVD), among all participants and men and women separately. Both cases and controls were included as the basis for division into seven percentiles where the one studied is compared to the rest.

	ALL (210/622)		MALES (139/415)		FEMALES (71/207)	
Anti-OxPS	Crude	Adjusted*	Crude	Adjusted*	Crude	Adjusted*
< = 10%	1.57(0.96–2.56)	1.55 (0.93–2.57)	1.95 (1.14–3.35)	1.96 (1.12–3.46)	0.58 (0.16–2.14)	0.57 (0.16–2.10)
< = 25%	1.31 (0.91–1.89)	1.29 (0.89–1.88)	1.66 (1.08–2.55)	1.65 (1.06–2.59)	0.70 (0.33–1.49)	0.72 (0.33–1.56)
< = 33%	1.23 (0.88–1.74)	1.25 (0.87–1.78)	1.56 (1.03–2.37)	1.64 (1.06–2.53)	0.71 (0.36–1.38)	0.69 (0.34–1.38)
>50%	0.85 (0.61–1.17)	0.83 (0.60–1.17)	0.81 (0.55–1.20)	0.76 (0.50–1.15)	0.93 (0.52–1.64)	0.96 (0.53–1.73)
>66%	0.73 (0.52–1.03)	0.71 (0.50–1.01)	0.74 (0.49–1.14)	0.67 (0.43–1.06)	0.71 (0.39–1.27)	0.76 (0.41–1.40)
>75%	0.63 (0.42–0.95)	0.60 (0.40–0.91)	0.57 (0.34–0.96)	0.50 (0.29–0.87)	0.74 (0.40–1.38)	0.74 (0.39–1.41)
>90%	0.78 (0.44–1.38)	0.70 (0.39–1.27)	0.50 (0.23–1.11)	0.43 (0.19–0.98)	1.54 (0.63–3.76)	1.61 (0.62–4.23)

*adjustment for smoking, BMI, type II diabetes, hypercholesterolaemia and hypertension

**Table 6:** Association between levels of IgM anti-OxPS and risk for stroke, among all participants and men and women separately. Both cases and controls were included as the basis for division into seven percentiles where the one studied is compared to the rest.

	ALL (49/144)		MALES (25/74)		FEMALES (24/70)	
Anti-OxPS	Crude	Adjusted*	Crude	Adjusted*	Crude	Adjusted*
< = 10%	2.18 (0.72–6.62)	2.51 (0.75–8.42)	3.00 (0.89–10.06)	3.40 (0.80–14.44)	N/A	
< = 25%	2.50 (1.06–5.89)	3.10 (1.23–7.78)	3.30 (1.08–10.12)	5.30 (1.37–20.5)	1.56 (0.36–6.68)	1.47 (0.31–6.93)
< = 33%	1.23 (0.57–2.61)	1.41 (0.63–3.16)	2.11 (0.77–5.75)	3.68 (1.00–13.55)	0.47 (0.10–2.15)	0.43 (0.09–2.00)
>50%	0.86 (0.42–1.76)	0.71 (0.33–1.54)	0.71 (0.26–1.95)	0.43 (0.13–1.47)	1.05 (0.37–3.01)	1.11 (0.36–3.48)
>66%	0.77 (0.36–1.63)	0.65 (0.29–1.47)	0.61 (0.20–1.90)	0.46 (0.13–1.65)	0.94 (0.34–2.58)	0.91 (0.30–2.78)
>75%	1.10 (0.49–2.53)	0.94 (0.39–2.26)	0.96 (0.26–3.55)	0.89 (0.20–3.88)	1.22 (0.42–3.53)	1.14 (0.38–3.48)
>90%	1.00 (0.28–3.57)	0.75(0.19–2.95)	N/A		2.72 (0.56–13.19)	2.35 (0.40–13.96)

*adjustment for smoking, BMI, type II diabetes, hypercholesterolaemia and hypertension

There were no significant associations between IgM anti-OxPS levels and CVD-manifestations among women. However, among men, associations were significant, also implicating IgM anti-OxPS as a novel protection marker. The 3^rd^ quartile compared to the highest, 4^th^, had OR 2.04 (CI 1.08–2.86). 2^nd^ and 1^st^ had similar associations, with 1^st^ reaching significance 2.47 (CI 1.30–4.72) as compared to the highest, 4^th^. Associations for relatively few stroke cases did not reach statistical significance, though the OR was very high for men, when lowest and highest quartiles were compared (8.85). However, for MI/angina, the associations reached significance: the 3^rd^ quartile compared to the highest, 4^th^ had OR 2.31 (CI 1.30–4.11). 1^st^ and 2^nd^ had similar and significant associations, as compared to 4^th^ (Table 3).

Further analyses indicate a negative association with high risk for those with high IgM anti-oxPS measures and a positive association could also be seen in some aspects of those having low IgM anti-OxPS levels, which we present in Tables 5 and 6. This association reached significance when the highest 25th percentile was compared to those below (crude: OR 0.63 and CI 0.42–0.95; adjusted for smoking, BMI, diabetes mellitus type II, hypercholesterolaemia, and high blood pressure: OR 0.60, CI 0.40–0.91.

For men these associations were more pronounced, which was not the case among women where there were no significant associations. Among men, at low levels we observed an increased risk after adjustment for the above mentioned confounders: below 10th percentile: OR 1.96, CI (1.12–3.46); 25th: OR 1.65, CI (1.06–2.59); 33rd: OR 1.64, CI (1.06–2.59).

Above the median, the inverse association was significant: above 75th: OR 0.50, CI (0.29–0.87) and above 90th: OR 0.43, CI (0.19–0.98).

When stroke was analysed separately there were significant associations (Table 6). At low levels (below 25^th^ and 33^rd^ percentile respectively), IgM anti-OxPS was significantly associated with increased risk for stroke: OR 5.30, CI (1.37–10.5) and OR 3.68, CI (1.00–13.5).

### Uptake of apoptotic cells by macrophages

**Fig 2:**
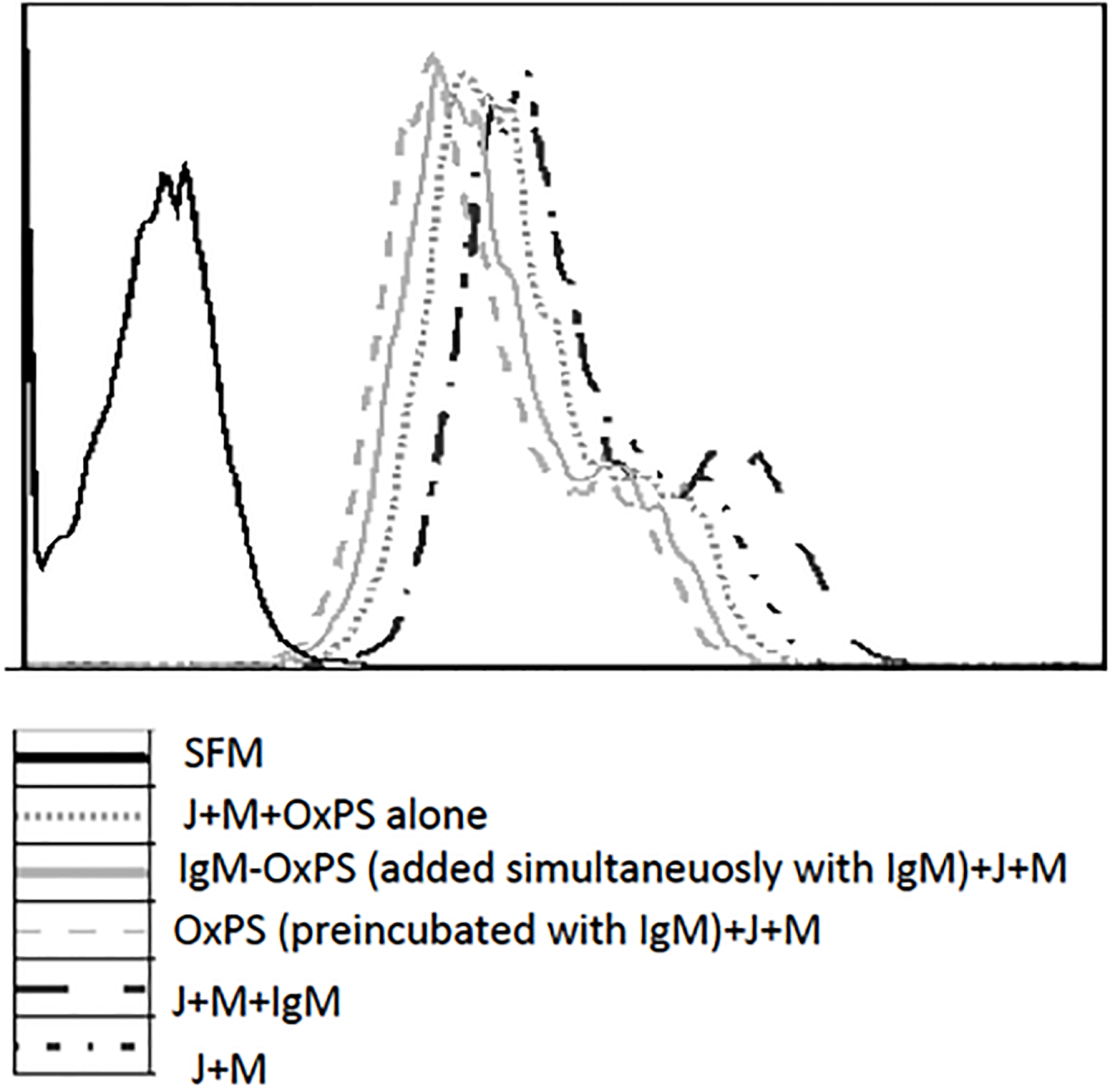
Effect on macrophage-mediated uptake of apoptotic cells. Apoptotic jurkat cells were co-cultured with macrophages 5:1 with or without IgM, IgM which was either preincubated for 90 minutes or simultaneously added in the cell culture system as indicated. Phagocytosis assay was analyzed by flow cytometry. Phagocytosis of apoptotic cells was increased by total IgM but decreased by oxPS. Preincubation or simultaneous incubation of IgM with OxPS added to the cell culture systems further decreased the uptake of apoptotic cells. SFM = serum free medium; J = Jurkat-cells; M = Macrophages; OxPS = oxidized phosphatidylserine

To investigate the role of IgM anti-OxPS in phagocytosis of apoptotic cells, macrophages were co-cultured with apoptotic Jurkat cells. Phagocytosis of apoptotic cells was increased by total IgM. Pre-incubation or simultaneous incubation of IgM with OxPS added to the cell culture systems decreased the uptake of apoptotic cells as compared to IgM alone. Further, OxPS also decreased uptake of apoptotic cells by itself (Fig 2). In three independent experiments (mean and SD), the proportion of cells which were taken up by macrophages increased from 50.7% ±1.2 to 59.0% ± 1.0 when IgM was added to Jurkat cells and macrophages but decreased to 26.3%± 3.8 when pre-incubated with oxPS and with 31.0% ± 5.0 when oxPS was added at the same time as IgM. OxPS in itself inhibited uptake with the proportion of cells taken up by macrophages at 32.3% ± 3.5. These changes were all significant as compared to Jurkat cells + macrophages alone (p<0.05).

## Discussion

This study demonstrates for the first time that IgM antibodies against OxPS are inversely associated with CVD and could thus be protection markers. IgM anti-OxPS were significantly decreased among 60-year olds who develop CVD within a 5–7 year follow-up period.

If divided into quartiles, with the highest set as 4^th^, having anti-OxPS values in the 3^rd^ quartile compared to the 4^th^ was a significant protection marker, and similar associations were present when lowest quartile was compared to the highest. Associations among men were even stronger, but there were no associations for women. There were relatively few stroke cases and there were no significant associations with anti-OxPS as analysed with highest quartile compared with lower ones, respectively though the OR was high when highest and lowest were compared. However, for MI/angina, this was the case: the lower quartiles compared with the highest, 4^th^ were all significantly increased. IgM anti-OxPS is thus a novel protection marker for CVD, especially among men, and specifically for MI/angina.

Further analyses comparing values above or below different cut-offs also gave interesting results. High levels of IgM anti-OxPS (above 75th percentile) were associated with a significantly decreased risk. Again, it is interesting to note that there were no significant associations when women were studied separately. Whether this is due to real differences between men and women in this age group in relation to IgM anti-OxPS or is more related to the lower number of women who developed CVD during the follow up period is as yet not clear. The possibility that this type of antibodies play another role at higher ages for women, could be considered in novel studies.

However, among men, low levels were associated with an almost 2-fold risk (at the lowest decile) and significant effects were also seen at the lowest 25th and 33rd percentiles. High levels above 75th and 90th were associated with a 2-times risk reduction.

When stroke was analysed separately, there was a striking association seen with a more than 5-fold increased risk at the lowest 25th percentile compared with the rest.

These significant associations were independent of other risk markers.

We recently reported a negative association with atherosclerosis and potentially vulnerable plaques in systemic lupus erythematosus (SLE) where the prevalence of atherosclerotic plaques and risk of CVD is very high[16]. Further, we reported that anti-OxPS is negatively associated with atherosclerosis progression among hypertensives[17]. The findings reported herein are thus in line with these recent findings.

PS is an important”eat me” signal to phagocytes and is thus a DAMP (danger-associated molecular pattern) which is externalized on cell membranes during cellular stress, apoptotis or necrosis. This occurs through an enzymatically controlled flip-flop process. This is generally seen as being of importance for clearance of apoptotic cells and cell debris. Dysfunctional clearance could play a role in chronic inflammatory diseases, especially in SLE this has been much discussed [19].

Also in atherosclerosis, defective clearance of dead cells in the necrotic core of atherosclerotic plaques has been implicated and is related to increased inflammation in and progression of atherosclerotic plaques in experimental models [20].

PS is easily oxidized, and it has been demonstrated that such oxPS could be of great importance mediating macrophage recognition and engulfment of apoptotic cells, more so than native PS[21]. Extra-mitochondrial cytochrome c could catalyze apoptosis-associated PS oxidation[22]. Further, OxPS is a ligand for the scavenger receptor CD36[13].

Our previous studies also indicate that IgM antibodies against other DAMPs, namely phosphorylcholine (PC) and oxidized cardiolipin (OxCL) have similar associations to development of CVD as anti-OxPS also in this patient cohort [11, 18, 23], though these antibodies are distinct entities, and their profiles as protective markers also differ [17, 24, 25].

We have previously demonstrated that anti-OxPS and anti-OxCL are not traditional thrombogenic anti-phospholipid antibodies and are not dependent on plasma co-factors as beta2-glycoprotein I which is the case with the traditional aPL, which are most likely recognizing CL or PS (or sometimes other phospholipids like phosphatidylcholine) in complex with co-factors. These phospholipids may also have undergone some oxidation, but not heavy oxidation as herein[24].

Uptake and clearance of dead cells by phagocytic cells as macrophages and dendritic is a major mechanism to maintain homeostasis. This is mediated by exposure of”eat-me” signals including PS on cell membranes. During recent years, it has become clear that oxidation of PS could be a major factor in this process. OxPS is a major ligand for uptake by macrophages of apoptotic cells[12] and oxidation of PS on apoptotic cells is abundant and has recently been quantified[26]. One mechanism by which PS could be oxidized is through phospholipase A2[27], which promotes such processes also lipoproteins.

Inefficient clearance of dead cells has been much discussed as a potential cause of SLE, which could create a proinflammatory state and also unwanted immune reactions (as in SLE) to targets exposed in a non-physiological way[28].

Accumulation of dead cells in the necrotic core of atherosclerotic plaques is also a major feature of atherosclerosis, though appears be clearly less studied than other components of plaques, especially oxLDL. The notion that defective clearance could influence atherosclerosis is supported by animal data where this can cause progression of atherosclerosis and also increased inflammation in plaques [20].

To identify mechanisms which could explain the protective role of IgM anti-OxPS, we studied both oxPS, total IgM (a pool of human IgM from many donors) and pre-incubation with oxPS of IgM (which decreases anti-OxPS[24]). Our data indicate that IgM per se increases phagocytosis of apoptotic cells, while pre-incubation or simultaneous incubation of IgM with oxPS decreases this effect by IgM. Further, in line with previous experiments using oxPS-containing liposomes[12], oxPS could also compete out uptake of apoptotic cells. These findings indicate that IgM anti-OxPS could be not only a protection marker, but also contribute to clearance of stressed, dying and dead cells from atherosclerotic plaques. Having low levels could predispose to fast atherosclerosis development and ensuing CVD due to a less effective clearance capacity. Further studies of the structural properties and details of the antigen are warranted to develop IgM anti-OxPS as a protection marker.

There were striking associations between low levels of anti-OxPS and increased risk of stroke—a more than five times increased risk was suggested for values below the 25^th^ percentile value as compared to higher values. The possibility that defective clearance of dead cells could be especially important in stroke (and carotid atherosclerosis and plaque vulnerability) should be further explored.

Taken together, our findings indicate that IgM anti-OxPS is a CVD risk marker at lower levels and a protection marker for CVD at higher levels. Low levels of these antibodies could be an underlying mechanism in CVD and atherosclerosis by a low capacity to clear dead cells from atherosclerotic plaques. These findings could thus have both therapeutic and diagnostic/risk marker evaluation implications.

## References

[1] Frostegard J, Ulfgren AK, Nyberg P, Hedin U, Swedenborg J, Andersson U, et al Cytokine expression in advanced human atherosclerotic plaques: dominance of pro-inflammatory (Th1) and macrophage-stimulating cytokines. Atherosclerosis. 1999;145(1):33–43. Epub 1999/07/31. 10428293 10.1016/s0021-9150(99)00011-8

[2] Frostegard J. Immunity, atherosclerosis and cardiovascular disease. BMC Med. 2013;11(1):117. Epub 2013/05/03. 23635324 10.1186/1741-7015-11-117 PMC3658954

[3] Ridker PM, Stampfer MJ, Rifai N. Novel risk factors for systemic atherosclerosis: a comparison of C-reactive protein, fibrinogen, homocysteine, lipoprotein(a), and standard cholesterol screening as predictors of peripheral arterial disease. Jama. 2001;285(19):2481–5. 11368701 10.1001/jama.285.19.2481

[4] Kaptoge S, Seshasai SR, Gao P, Freitag DF, Butterworth AS, Borglykke A, et al Inflammatory cytokines and risk of coronary heart disease: new prospective study and updated meta-analysis. Eur Heart J. 2014;35(9):578–89. Epub 2013/09/13. 10.1093/eurheartj/eht367 24026779 PMC3938862

[5] Libby P, Ridker PM, Hansson GK. Progress and challenges in translating the biology of atherosclerosis. Nature. 2011;473(7347):317–25. Epub 2011/05/20. 10.1038/nature10146 21593864

[6] Zalewski A, Nelson JJ, Hegg L, Macphee C. Lp-PLA2: a new kid on the block. Clin Chem. 2006;52(9):1645–50. 10.1373/clinchem.2006.070672 16873290

[7] Frostegard J. Immunity, atherosclerosis and cardiovascular disease. BMC Med. 11:117 Epub 2013/05/03. 10.1186/1741-7015-11-117 23635324 PMC3658954

[8] Frostegard J. Low level natural antibodies against phosphorylcholine: a novel risk marker and potential mechanism in atherosclerosis and cardiovascular disease. Clin Immunol. 2010;134(1):47–54. Epub 2009/09/15. 10.1016/j.clim.2009.08.013 19748321

[9] Su J, Hua X, Concha H, Svenungsson E, Cederholm A, Frostegard J. Natural antibodies against phosphorylcholine as potential protective factors in SLE. Rheumatology (Oxford). 2008;47(8):1144–50. Epub 2008/06/05. 18522961 10.1093/rheumatology/ken120

[10] Anania C, Gustafsson T, Hua X, Su J, Vikstrom M, de Faire U, et al Increased prevalence of vulnerable atherosclerotic plaques and low levels of natural IgM antibodies against phosphorylcholine in patients with systemic lupus erythematosus. Arthritis Res Ther. 2010;12(6):R214 Epub 2010/11/26. 10.1186/ar3193 21092251 PMC3046524

[11] Su J, Hua X, Vikstrom M, Leander K, Gigante B, Hellenius ML, et al Low levels of IgM antibodies to oxidized cardiolipin increase and high levels decrease risk of cardiovascular disease among 60-year olds: a prospective study. BMC Cardiovasc Disord. 2013;13(1):1. Epub 2013/01/09. 23294904 10.1186/1471-2261-13-1 PMC3560105

[12] Kagan VE, Gleiss B, Tyurina YY, Tyurin VA, Elenstrom-Magnusson C, Liu SX, et al A role for oxidative stress in apoptosis: oxidation and externalization of phosphatidylserine is required for macrophage clearance of cells undergoing Fas-mediated apoptosis. J Immunol. 2002;169(1):487–99. Epub 2002/06/22. 12077280 10.4049/jimmunol.169.1.487

[13] Greenberg ME, Sun M, Zhang R, Febbraio M, Silverstein R, Hazen SL. Oxidized phosphatidylserine-CD36 interactions play an essential role in macrophage-dependent phagocytosis of apoptotic cells. J Exp Med. 2006;203(12):2613–25. Epub 2006/11/15. 10.1084/jem.20060370 17101731 PMC2118161

[14] Halldin M, Rosell M, de Faire U, Hellenius ML. The metabolic syndrome: prevalence and association to leisure-time and work-related physical activity in 60-year-old men and women. Nutr Metab Cardiovasc Dis. 2007;17(5):349–57. 10.1016/j.numecd.2006.01.002 17562572

[15] Carlsson AC, Wandell PE, de Faire U, Hellenius ML. Risk factors associated with newly diagnosed high blood pressure in men and women. Am J Hypertens. 2008;21(7):771–7. 10.1038/ajh.2008.167 18437125

[16] Su J, Frostegard AG, Hua X, Gustafsson T, Jogestrand T, Hafstrom I, et al Low Levels of Antibodies Against Oxidized but not Nonoxidized Cardiolipin and Phosphatidylserine Are Associated with Atherosclerotic Plaques in Systemic Lupus Erythematosus. J Rheumatol. 2013. Epub 2013/09/17. 10.3899/jrheum.121173 24037548

[17] Frostegard AG, Su J, Hua X, Vikstrom M, de Faire U, Frostegard J. Antibodies against Native and Oxidized Cardiolipin and Phosphatidylserine and Phosphorylcholine in Atherosclerosis Development. PLoS One. 2014;9(12):e111764 10.1371/journal.pone.0111764 25473948 PMC4256296

[18] de Faire U, Su J, Hua X, Frostegard A, Halldin M, Hellenius ML, et al Low levels of IgM antibodies to phosphorylcholine predict cardiovascular disease in 60-year old men: effects on uptake of oxidized LDL in macrophages as a potential mechanism. J Autoimmun. 2010;34(2):73–9. Epub 2009/09/04. 10.1016/j.jaut.2009.05.003 19726160

[19] Jitkaew S, Witasp E, Zhang S, Kagan VE, Fadeel B. Induction of caspase- and reactive oxygen species-independent phosphatidylserine externalization in primary human neutrophils: role in macrophage recognition and engulfment. J Leukoc Biol. 2009;85(3):427–37. Epub 2008/12/25. 10.1189/jlb.0408232 19106181 PMC2653945

[20] Silvestre-Roig C, de Winther MP, Weber C, Daemen MJ, Lutgens E, Soehnlein O. Atherosclerotic plaque destabilization: mechanisms, models, and therapeutic strategies. Circ Res. 2014;114(1):214–26. Epub 2014/01/05. 10.1161/CIRCRESAHA.114.302355 24385514

[21] Arroyo A, Modriansky M, Serinkan FB, Bello RI, Matsura T, Jiang J, et al NADPH oxidase-dependent oxidation and externalization of phosphatidylserine during apoptosis in Me2SO-differentiated HL-60 cells. Role in phagocytic clearance. J Biol Chem. 2002;277(51):49965–75. Epub 2002/10/12. 10.1074/jbc.M204513200 12376550

[22] Jiang J, Kini V, Belikova N, Serinkan BF, Borisenko GG, Tyurina YY, et al Cytochrome c release is required for phosphatidylserine peroxidation during Fas-triggered apoptosis in lung epithelial A549 cells. Lipids. 2004;39(11):1133–42. Epub 2005/02/25. 15726829 10.1007/s11745-004-1340-1

[23] Sjoberg BG, Su J, Dahlbom I, Gronlund H, Wikstrom M, Hedblad B, et al Low levels of IgM antibodies against phosphorylcholine-A potential risk marker for ischemic stroke in men. Atherosclerosis. 2009;203(2):528–32. 10.1016/j.atherosclerosis.2008.07.009 18809177

[24] Su J, Frostegard AG, Hua X, Gustafsson T, Jogestrand T, Hafstrom I, et al Low levels of antibodies against oxidized but not nonoxidized cardiolipin and phosphatidylserine are associated with atherosclerotic plaques in systemic lupus erythematosus. J Rheumatol. 2013;40(11):1856–64. 10.3899/jrheum.121173 24037548

[25] Fiskesund R, Stegmayr B, Hallmans G, Vikstrom M, Weinehall L, de Faire U, et al Low levels of antibodies against phosphorylcholine predict development of stroke in a population-based study from northern Sweden. Stroke. 2010;41(4):607–12. Epub 2010/02/13. 10.1161/STROKEAHA.109.558742 20150554

[26] Fabisiak JP, Tyurina YY, Tyurin VA, Kagan VE. Quantification of selective phosphatidylserine oxidation during apoptosis. Methods Mol Biol. 2005;291:449–56. 15502241 10.1385/1-59259-840-4:449

[27] Tyurin VA, Yanamala N, Tyurina YY, Klein-Seetharaman J, Macphee CH, Kagan VE. Specificity of lipoprotein-associated phospholipase A(2) toward oxidized phosphatidylserines: liquid chromatography-electrospray ionization mass spectrometry characterization of products and computer modeling of interactions. Biochemistry. 2012;51(48):9736–50. 10.1021/bi301024e 23148485 PMC3567262

[28] Nagata S, Hanayama R, Kawane K. Autoimmunity and the clearance of dead cells. Cell. 2010;140(5):619–30. Epub 2010/03/10. 10.1016/j.cell.2010.02.014 20211132

